# Comparative evaluation of marginal bone loss and prosthetic success in immediate versus delayed implant loading protocols: A radiographic study

**DOI:** 10.6026/973206300220042

**Published:** 2026-01-31

**Authors:** Nakul Chaudhary, Chinmay Sharadchandra Dave, Prachi Sharma, Devisree R.V, Gunjan Gupta, Shailendra Pal Singh

**Affiliations:** 1 Department of Oral & Maxillofacial Surgery, Teerthanker Mahaveer Dental College & Research Centre, Moradabad, Uttar Pradesh, India; 2 Department of Orthodontics and Dentofacial Orthopaedics, Government Dental College and Hospital, Jamnagar, Gujarat, India; 3 Department of Neurology, All India Institute of Medical Sciences, Delhi, India; 4 Department of Periodontology, Azeezia College of Dental Sciences and Research, Kollam, Kerala, India; 5 Department of Periodontology, Maharana Pratap College of Dentistry and Research Centre, Gwalior, Madhya Pradesh, India

**Keywords:** Dental implants, immediate loading, marginal bone loss, prosthetic success

## Abstract

The comparison of the effects of immediate and delayed implant loading protocols on marginal bone loss and prosthetic success over a 12-month period is highly relevant. Therefore, it is of interest to compare the effects of immediate and delayed implant loading protocols on marginal bone loss and prosthetic success over 12 months. Hence, a total of 100 participants were randomly assigned to either the immediate or delayed loading group. Results showed that the delayed loading group experienced significantly less marginal bone loss and better bone preservation. Implant survival and prosthetic success rates were high in both groups, with no significant differences observed. Thus, we show that while immediate loading provides aesthetic advantages, delayed loading may offer better long-term bone preservation.

## Background:

Dental implants have revolutionized the field of prosthodontics, providing a reliable and durable solution for replacing missing teeth. Their success is influenced by several factors, including the type of implant, the quality of the surrounding bone, surgical technique, and the loading protocol [1]. One of the most debated aspects of dental implantology is the timing of the implant loading - whether the prosthetic restoration is placed immediately after implant placement (immediate loading) or after a healing period (delayed loading) [2]. The loading protocol can significantly impact the long-term stability of the implant and the surrounding bone, which are critical to the overall success of the treatment [3]. Immediate loading involves the placement of a provisional or final restoration on the implant within 48 hours of its surgical placement. This approach is gaining popularity due to its potential to shorten treatment time and improve patient satisfaction by offering immediate esthetics and function [4]. Immediate loading is particularly beneficial for patients with limited time or those seeking a faster solution. However, the primary concern with this technique is whether the implant achieves sufficient primary stability to withstand functional loads. If the implant lacks stability, premature loading may lead to increased stress on the surrounding bone, potentially causing bone resorption and compromising osseointegration [5]. In contrast, delayed loading involves waiting several months after implant placement before applying the prosthetic restoration. This allows time for osseointegration, where the implant integrates with the surrounding bone, providing a stable foundation for the prosthesis [6]. Delayed loading is considered the gold standard due to its potential to enhance implant stability and reduce the risk of bone loss. The longer healing time associated with delayed loading, however, may be seen as a disadvantage, particularly in terms of patient satisfaction and treatment duration. Moreover, the delayed approach may not be suitable for patients seeking immediate functional or aesthetic outcomes [7]. The debate over which loading protocol is superior remains ongoing, with studies showing mixed results regarding the impact of each protocol on marginal bone loss and implant success. Marginal bone loss, in particular, is a critical factor in the long-term success of dental implants. Excessive bone loss can lead to implant failure, reduced esthetics, and compromised prosthetic function [8]. Some studies suggest that immediate loading may lead to greater bone loss around the implant, particularly in the early stages of healing when osseointegration is not yet complete. Conversely, delayed loading is believed to allow for more controlled osseointegration and better bone preservation. Radiographic assessments play a key role in evaluating marginal bone loss and implant stability over time [9]. Therefore, it is of interest to determine the comparative effects of immediate and delayed loading on marginal bone loss and prosthetic success, providing valuable insights that can help optimize clinical practices in implant dentistry.

## Methodology:

This study aimed to evaluate the comparative effects of immediate versus delayed loading protocols on marginal bone loss and prosthetic success in dental implants. A prospective, randomized controlled trial (RCT) design was used to compare the outcomes of the two loading protocols. The primary outcomes of the study included marginal bone loss, while secondary outcomes focused on implant survival, prosthetic success, and functional and aesthetic outcomes. Radiographic imaging was used to measure the marginal bone loss around the implants over time. The study was conducted in patients requiring dental implants for the rehabilitation of missing teeth. Inclusion criteria included adults aged 18-70 years, patients with fully healed edentulous sites, and adequate bone volume (≥5 mm height and ≥6 mm width). Additionally, participants had no systemic contraindications for surgery (e.g., uncontrolled diabetes, osteoporosis, immunocompromised conditions) and were willing to comply with the study protocol, including follow-up visits. Patients who were smokers (more than 10 cigarettes per day), those who had undergone radiotherapy in the head or neck area, or had a history of implant failure or infection, were excluded from the study. The sample size for the study was calculated using G*Power software to detect significant differences in marginal bone loss between the two groups. Based on an effect size of 0.5, an alpha level of 0.05, and a power of 0.80, it was determined that 50 participants per group (immediate loading and delayed loading) would be required, resulting in a total sample size of 100 participants. This calculation assumed that the primary outcome of marginal bone loss would exhibit a medium effect size between the two groups. The final sample size ensured adequate statistical power to detect differences between the two groups. In the immediate loading group, implants were placed, and a provisional restoration was attached within 48 hours of surgery. This provisional restoration was designed to allow functional occlusion. In the delayed loading group, implants were placed and allowed to heal for 3-6 months before a prosthetic restoration was applied. Both groups received the same type of titanium screw-type implants, and the same prosthetic designs were used for the final restorations. Radiographic evaluations were performed using periapical radiographs at multiple time points to assess marginal bone loss. Radiographs were taken immediately post-surgery, at 3 months, 6 months, and 12 months after implant placement. Marginal bone loss was measured by assessing the distance between the implant shoulder and the first visible bone-to- implant contact using calibrated radiographic measurement tools. Prosthetic success was evaluated based on several criteria, including implant survival, restoration stability, and functional and aesthetic outcomes. Implant survival was determined by the number of implants still in place at the end of the study. Restoration stability was assessed by checking for any signs of loosening, fracture, or failure. Functional outcomes were evaluated by patient-reported measures of their ability to chew, speak, and perform other daily activities with the restoration. Aesthetic outcomes were assessed using a visual analog scale (VAS) by both the treating dentist and patients. Data analysis involved the use of descriptive statistics to summarize demographic and baseline characteristics, as well as continuous variables such as marginal bone loss and implant survival rates. Inferential statistics were used to compare outcomes between the two groups. An independent t-test was employed to compare the mean marginal bone loss between the immediate and delayed loading groups at each time point. A chi-square test was used to compare the proportion of implant survival and prosthetic success between the two groups. Repeated measures ANOVA were used to assess changes in marginal bone loss over time (baseline, 3 months, 6 months, and 12 months) between the two groups. A significance level of p < 0.05 was set for all statistical tests. The study was conducted in compliance with ethical guidelines, and approval was obtained from the institutional review board (IRB). Written informed consent was obtained from all participants, ensuring that they understood the nature of the study, the risks, and the benefits of participation. Patient confidentiality and anonymity were maintained throughout the study.

##  Results:

The results of this study were based on a comparative evaluation of immediate versus delayed loading protocols for dental implants. A total of 100 participants (50 in each group) were included, and data were collected over 12 months. The primary outcome measured was marginal bone loss, with secondary outcomes focusing on implant survival, prosthetic success, and functional and aesthetic outcomes. The following results summarize the key findings in terms of radiographic assessment, implant survival, and prosthetic success. The demographic characteristics of the participants are summarized in Table 1. No significant differences were observed between the two groups in terms of age, gender, or baseline bone quality (p > 0.05) (Table 1). Marginal bone loss was measured at baseline, 3 months, 6 months, and 12 months following implant placement. The results indicated that both groups experienced a gradual decrease in marginal bone height over time. However, the immediate loading group showed a significantly higher rate of bone loss at 3 and 6 months compared to the delayed loading group (p < 0.05). By 12 months, the delayed loading group had significantly less bone loss compared to the immediate loading group (p = 0.02) (Table 2). The immediate loading group showed greater marginal bone loss over time, particularly at the 3-month and 6-month time points. In contrast, the delayed loading group demonstrated better preservation of marginal bone levels over the 12 months (Table 3). Implant survival was assessed based on whether the implants remained functional throughout the study period. At 12 months, the implant survival rate was high in both groups, with 98% survival in the immediate loading group and 96% survival in the delayed loading group. No statistically significant difference was found between the two groups in terms of implant survival (p = 0.652). Both immediate and delayed loading protocols demonstrated excellent implant survival, with only minor failures observed, all of which were unrelated to bone integration. The absence of complications such as loosening, fracture, or failure of the restoration defines prosthetic success. At 12 months, 94% of the implants in the immediate loading group had stable prosthetic restorations, compared to 90% in the delayed loading group. The differences between the two groups in terms of prosthetic success were not statistically significant (p = 0.395) (Table 4). Both groups exhibited high rates of prosthetic success, with a minor percentage of failures due to mechanical issues, most of which were resolved promptly. Functional and aesthetic outcomes were assessed using patient- reported measures and VAS. Patients in both groups reported significant improvements in their ability to chew, speak, and perform daily activities post-surgery. However, the immediate loading group reported slightly higher satisfaction in terms of aesthetics at 3 months, with a VAS score of 8.2 ± 1.3 compared to 7.6 ± 1.5 in the delayed loading group (p = 0.048). At 12 months, both groups reported similar levels of satisfaction (Table 5). At the 12-month follow-up, both groups showed high levels of satisfaction, indicating that both loading protocols led to functional and aesthetic success.

## Discussion:

In this study comparing immediate versus delayed loading protocols, our findings indicate that while both approaches achieve high implant survival rates and good prosthetic success, clinically relevant differences in marginal bone loss emerged. The delayed loading group exhibited better preservation of peri-implant bone over the 12-month follow-up, supporting the notion that allowing for an extended osseointegration period may enhance bone stability. This aligns with prior research showing that delayed (or conventional) loading reduces peri-implant bone loss compared to immediate loading. For example, a systematic review by Rojas-Rojas *et al.* (2024) [10] found that while immediate loading has comparable implant survival, it shows a trend toward higher marginal bone loss. Our results also reflect the complexity of interpreting "superiority" of loading protocols: implant survival rates did not significantly differ between groups, which echoes findings from the study by Patel *et al.* (2024) [10], such as the meta-analysis of immediate vs delayed placement showing no significant difference in survival (risk ratio ≈ 0.99). This suggests that, from a survival standpoint, both protocols are viable in suitable cases, but bone-level changes may provide finer discrimination of long-term outcomes. In the present study, the immediate loading group exhibited slightly greater early bone loss (at 3 and 6 months) compared to the delayed group, which is consistent with biomechanical concerns: early loading may introduce micromovement or increased mechanical stresses before complete osseointegration, leading to marginal bone remodeling or resorption. A RCT focused on unsplinted mandibular implant-retained overdentures found that marginal bone loss around immediately loaded implants was comparable to delayed loading (weighted mean differences were not statistically significant) in that specific context. This finding suggests that in certain specialized prosthetic scenarios, the loading time difference may be less critical for bone loss, as indicated by Kutkut *et al.* (2019) [11]. However, our study involved standard implants and restorations, where the difference was more evident. Another 10-year RCT in the aesthetic zone found a negligible difference in marginal bone level changes between immediate vs delayed provisionalisation (-0.47mm vs -0.58mm), showing that with appropriate case selection, immediate loading can yield stable bone levels. The clinical implications of our findings are that while immediate loading offers patient-centric benefits (shorter treatment time, immediate function, and esthetics), clinicians must consider bone quality, primary stability, occlusal scheme, and patient risk factors when selecting this protocol. The slightly greater bone loss observed in the immediate group suggests that delayed loading may be preferable in cases where maximal bone preservation is critical (e.g., aesthetic zone, thin biotype, compromised bone). Our findings reinforce that decision-making should be individualized rather than one size fits as shown elsewhere by Kourkoutis *et al.* (2025) [12].

## Conclusion:

We show that both loading protocols can achieve successful implant survival and prosthetic outcomes; however, delayed loading offers better marginal bone preservation in the first year. These differences should inform treatment planning and the selection of patient- specific protocols. Therefore, this study is essential to determine the comparative effects of immediate and delayed loading on marginal bone loss and implant stability.

## Figures and Tables

**Table 1:** Demographic characteristics of study participants

**Characteristic**	**Immediate Loading (n=50)**	**Delayed Loading (n=50)**	**p-value**
Age (mean ± SD)	46.2 ± 8.1	47.1 ± 7.6	0.492
Gender (M/F)	25/25	26/24	0.81
Bone Quality	3.5 ± 0.7	3.4 ± 0.6	0.687

**Table 2:** Marginal bone loss at different time points

**Time Point**	**Immediate Loading (mm)**	**Delayed Loading (mm)**	**p-value**
Baseline	0.0	0.0	-
3 months	1.12 ± 0.38	0.85 ± 0.30	0.042
6 months	1.34 ± 0.42	1.02 ± 0.33	0.017
12 months	1.56 ± 0.45	1.21 ± 0.38	0.02

**Table 3:** Implant survival at 12 months

**Group**	**Implants Survived**	**Implants Failed**	**Survival Rate (%)**	**p-value**
Immediate Loading	49	1	98%	0.652
Delayed Loading	48	2	96%	

**Table 4:** Prosthetic success at 12 months

**Group**	**Successful Restorations**	**Unstable Restorations**	**Success Rate (%)**	**p-value**
Immediate Loading	47	3	94%	0.395
Delayed Loading	45	5	90%	

**Table 5:** Functional and aesthetic outcomes (VAS Score)

**Time Point**	**Immediate Loading (VAS)**	**Delayed Loading (VAS)**	**p-value**
3 months	8.2 ± 1.3	7.6 ± 1.5	0.048
12 months	9.1 ± 0.8	9.0 ± 0.9	0.436
